# Procalcitonin determined at emergency department as an early indicator of progression to septic shock in patient with sepsis associated with ureteral calculi

**DOI:** 10.1590/S1677-5538.IBJU.2014.0465

**Published:** 2016

**Authors:** Young Hwii Ko, Yoon Seob Ji, Sin-Youl Park, Su Jin Kim, Phil Hyun Song

**Affiliations:** Department of Urology, College of Medicine, Yeungnam University, Daegu, Korea; Department of Emergency medicine, College of Medicine, Yeungnam University, Daegu, Korea; Department of Emergency medicine, Korea University Anam Hospital, Seoul, Korea

**Keywords:** Ureteral Calculi, Pyelonephritis, Shock, Septic

## Abstract

**Introduction:**

To investigate the role of initial procalcitonin (PCT) level as an early predictor of septic shock for the patient with sepsis induced by acute pyelonephritis (APN) secondary to ureteral calculi.

**Materials and Methods:**

The data from 49 consecutive patients who met criteria of sepsis due to APN following ureteral stone were collected and divided into two groups: with (n=15) or without (n=34) septic shock. The clinical variables including PCT level for this outcome were retrospectively compared by univariate analysis, followed by multivariable logistic regression model.

**Results:**

All subjects had hydronephrosis, and were hospitalized with the mean of 11.8 days (3–42 days). The mean size of the ureteral stones was 7.5mm (3–30mm), and 57% were located in upper ureter. At univariate analysis, patients with septic shock were significantly older, a higher proportion had hypertension, lower platelet count and serum albumin level, higher CRP and PCT level, and higher positive blood culture rate. Multivariate models indicated that lower platelet count and higher PCT level are independent risk factors (p=0.043 and 0.046, respectively). In ROC curve, the AUC was significantly wider in PCT (0.929), compared with the platelet count (0.822, p=0.004). At the cut-off of 0.52ng/mL, the sensitivity and specificity were 86.7% and 85.3%.

**Conclusion:**

Our study demonstrated elevated initial PCT levels as an early independent predictor to progress into septic shock in patients with sepsis associated with ureteral calculi.

## Introduction

Urinary tract infection (UTI) is the second most common infectious cause for hospitalization in aged people and one of the most common cause of antibiotics usage (1, 2). Patients with febrile UTI generally present with mild illness in primary care, but an estimated 41% of those with complicated acute pyelonephritis (APN) develop severe sepsis (3). Though overall mortality from APN is approximately 0.3%, when accompanied by septic shock, mortality increases dramatically (4). With bacteremia, the overall mortality rate of APN can be as high as 7.5% to 30% (4, 5). Given the high prevalence of UTIs, therefore, delay in diagnosis and treatment often results in a rapid progression with a lethal outcome. Though the positive bacterial culture has a major effect on the treatment, it is now generally accepted that the detection of bacteremia, requiring at least 24 to 48 hours from initial visit, is not a prerequisite for making the clinical diagnosis of sepsis (6). Thus, there is a need for strategies to identify the high risk patient earlier.

Procalcitonin (PCT) was recently introduced as a novel predictor for systemic infection. PCT is induced in the plasma of patients with severe bacterial or fungal infections or sepsis. PCT concentrations up to 1000ng/ml and above are observed during severe sepsis and septic shock. PCT concentrations are associated with the severity of multiple organ dysfunction syndrome secondary to systemic inflammation of infectious origin (7, 8). Randomized controlled trails had demonstrated efficacy in reduction of antibiotics usage (8), and FDA approved its use to assess the risk of critically ill patients progressing to severe sepsis. However, PCT levels may vary early during the development of sepsis. Also, it had been reported that the test's predictive power is only significant later in the patient's course (6, 9, 10). Moreover, most studies about the link between PCT and septic shock focused on generally ill patients with heterogeneous clinical conditions rather than a specific disease, compromising the predictability of PCT. Therefore, we investigated the role of PCT as an early predictor of progression to septic shock among patients with sepsis induced by APN secondary to ureteral calculi, which represents a significant portion of emergency department (ED) visits (11).

## Materials and Methods

### Patients and Data collection

Among 574 patients who visited the ED from January 2005 to June 2012 for clinical manifestations of APN following ureteral calculi, the data from 49 consecutive patients who met criteria of sepsis were collected, after approval of institutional reviewer board. The 49 patients were divided into two groups: with (n=15) or without (n=34) septic shock. While in the ED, the patient's age, sex, height and weight were recorded. Symptoms, prior medical or surgical history, and history of ureteral calculi were investigated. For the patient with possible febrile UTI, our routine laboratory protocol consisted of serum samples for white blood cell count, platelet count, creatinine, albumin, C-reactive protein (CRP), erythrocyte sedimentation rate (ESR), PCT and blood culture, and a urine culture, obtained at the time of admission to ED before commencing antimicrobial therapy. PCT levels were measured by using an enzyme-linked fluorescence assay (VIDAS® BRAHMS PCT assay; Biomerieux, Lyon, France). When there was clinical suspicion of ureteral calculi, our routine policy on the initial radiologic work ups was abdominal CT (with/without contrast enhancement) or ultrasonography. Based on this, the presence, location, and size of stone were identified. The diagnosis of APN was based on clinical manifestation, body temperature, and radiologic findings. Sepsis in this series was defined as systemic inflammatory response syndrome (SIRS), the presence of two or more of the following: abnormal body temperature, heart rate, respiratory rate or blood gas, and white blood cell count (12). Septic shock was defined as severe sepsis plus one of the following: mean blood pressure (BP) <60mmHg (<80mmHg for patients with known hypertension) after 40ml/kg of saline or the need for dopamine (5mg/kg/min) to maintain a mean BP>60mmHg (80mmHg for prior hypertension) (13). Patients were defined as with or without septic shock retrospectively, without knowledge of serum PCT levels, on the basis of the complete patient charts, results of microbiological cultures, and radiologic findings.

### Statistical analysis

The clinical variables of the two groups were initially compared by univariate analysis, using Student's t-test or Mann-Whitney U test for continuous variables and Pearson's chi-square tests for categorical variables. Covariates found to be associated with septic shock on univariate analysis at a level of significance p-value below 0.2 were eligible for inclusion in multivariable approach (14). Multivariable logistic regression models using forward conditional procedure were then conducted to identify independent predictors of septic shock. The ability of each variable to predict septic shock was evaluated by performing receiver operating characteristic (ROC) analyses, and the areas under the receiver operative characteristic curve (AUCs) were determined. For estimating the overall differential power of the several variables including biomarkers, we estimated ROC curve and AUCs. Based on ROC, points were assigned to calculate corresponding sensitivity and specificity. All data were collected using SPSS ver. 20.0 (SPSS, Inc., an IBM Company, Chicago, Illinois, USA). We rejected null hypotheses of no difference if p-values were less than. 05 in multivariate analysis and ROC curve.

## Results

Mean age was 67.2 years (range: 24–88 years), and 41 cases (83.7%) occurred in females. All cases had hydronephrosis, and nineteen (38.8%) patients were managed by percutaneous nephrostomy for urgent drainage. All patients were hospitalized and the mean hospital stay was 11.8 days (range: 3–42 days). The most common medical comorbidity was hypertension (28 patients, 57.1%) followed by diabetes mellitus (14 patients, 28.6%) and cerebrovascular accident (12 patients, 24.5%). Eleven patients (22.4%) had a prior history of ureteral calculi. In 28 patients (57.1%), the ureteral stones were located in upper ureter, while 21 patients (42.9%) had lower ureteral stones. The mean size of the stones was 7.47mm (range: 3–30mm).

**Figure 1:**
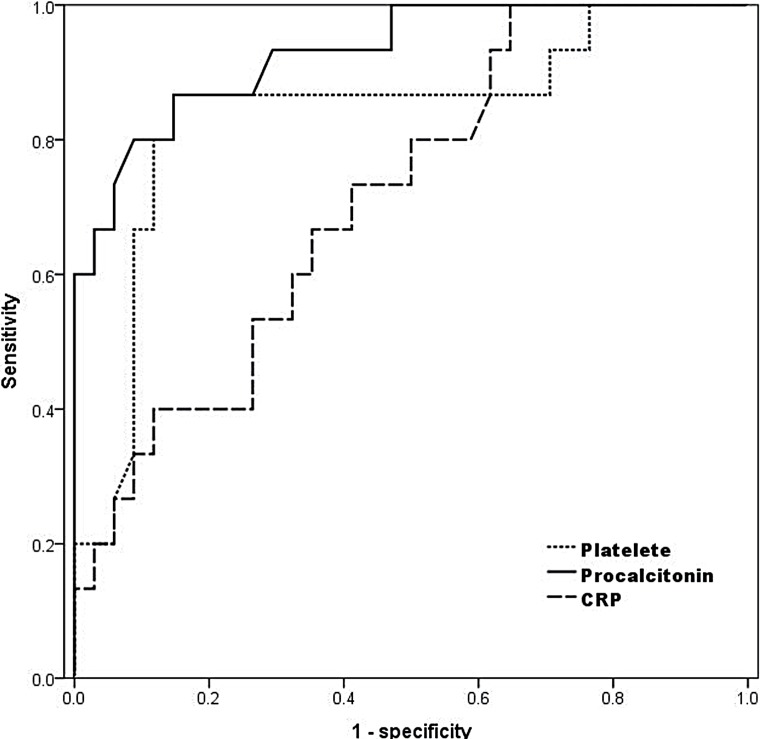
ROC curve for the prediction for septic shock.

In univariate analysis, patients with septic shock were significantly older, more likely to have hypertension, lower platelet count and serum albumin level, higher CRP and PCT level, and higher positive blood culture rate than the comparison patients (p=0.01, 0.002, <0.001, <0.001, 0.04, 0.005, and 0.02, respectively, Table-1). Radiologic characteristics of ureteral stone were not significantly different between groups. Multivariate analysis with the variables selected from the univariate analysis including leukocytosis (p=0.053) and stone size (p=0.103) indicated lower platelet count and higher PCT level as independent risk factors for septic shock (p=0.043 and 0.046, respectively). At ROC curve, AUC was significantly wider in PCT (0.929), compared with platelet count (0.822, p=0.004, Figure-1). At the cut-off of 0.52ng/mL, the sensitivity and specificity for septic shock at this point were 86.7% and 85.3% respectively.

**Table 1:** Patient demographics with outcomes from univariate analysis.

Characteristics (Mean±SD)		Total (n=49)	Septic shock		
			Positive (n=15)	Negative (n=34)	p-value
**Gender**					
	Male	8	2	6	
	Female	41	13	28	0.704
	Age	67.22±14.18	73.00±6.19	64.68±15.94	0.012
**BMI (kg/m^2^)**		24.43±5.51	23.47±3.00	24.86±6.30	0.300
**Medical comorbidity (%)**					
	HTN	28 (57.1)	13 (86.7)	15 (44.1)	0.002
	DM	14 (28.6)	7 (46.7)	9 (26.5)	0.098
	CVA	12 (24.5)	4 (26.7)	8 (23.5)	0.824
	MI	7 (14.3)	3 (20)	4 (11.7)	0.502
	WBC (/μL)	13390±5136	15207±6148	12589±4491	0.153
	Platelet (/μL)	177755±91415	104400±71467	210117±80725	<0.001
	Albumin (g/dL)	3.38±0.64	2.81±0.37	3.64±0.57	<0.001
	CRP (mg/dL)	9.51±10.28	14.46±11.38	7.32±9.10	0.043
	ESR (mm/H)	47.08±27.26	44.40±23.76	48.26±28.92	0.628
	Procalcitonin (μg/dL)	17.07±41.92	54.48±62.14	0.57±1.31	0.005
**Positive on blood culture (%)**		13 (26.5)	7 (46.6)	6 (17.6)	0.026
**Positive on urine culture (%)**		22 (44.9)	7 (46.6)	15 (44.1)	0.981
**Stone size (mm)**		7.47±4.28	6.27±2.46	8.00±4.81	0.103
**Drainage of urinary tract (%)**		19 (38.8)	7 (46.7)	12 (35.3)	0.451

## Discussion

Urolithiasis is a common cause of ED visits Average of 1.2 million patients per year were identified with the diagnosis of urolithiasis out of 120 million visits to the ED annually, with overall average admission rate of 19% (11). Though it may be asymptomatic, nearly 1 in 7 Americans suffer from urolithiasis during their lifetime (15), many of whom present with typical colicky pain at ED. Considering the increasing prevalence of infected urolithiasis of 15.5% and associated sepsis of 8.5% from recently updated data in comparison with a decade ago (16), we focused on sepsis caused by APN associated with ureteral calculi because it is a common sequel due to ureteral obstruction.

The outcomes from our series reveal several noteworthy findings. First, our data demonstrate the superiority of PCT in predicting the group at risk of septic shock among patients with sepsis associated with infected urolithiasis, in comparison with traditional widespread biomarkers for systemic infection. Although predictive values for clinical variables including PCT, CRP, albumin, thrombocytopenia, and bacteremia in univariate analysis are similar to published research, multivariate modeling selected only two factors, and the ROC curve demonstrated a significantly higher AUC for PCT than for thrombocytopenia.

The unique advantage of PCT in comparison with traditional biomarkers including CRP is its more rapid kinetics. PCT reacts faster than CRP both during an increase or decrease of inflammation (7). PCT increases within six hours after the initial stimulus and CRP does not increase significantly before twelve hours after onset. PCT primarily indicates systemic infection, not induced by local bacterial infection, viral infection, autoimmune and allergic disorder. PCT also accurately predicts the presence of bacteremia and bacterial load in patients with febrile UTI (14). Based on these characteristics, the availability of PCT immunoassays lessened the importance of CRP as a biomarker of sepsis. Indeed, in a previous multi-center study of patients with sepsis admitted to French intensive care units, a PCT-guided strategy for treating bacterial infections safely reduced antibiotic exposure compared to present guidelines (17). In a clinical pediatric study, PCT was not only useful in the early diagnosis of APN but also was highly associated with renal damage (18).

However, several investigators have questioned the diagnostic accuracy of PCT, as results have been inconsistent and variable (19, 20). For instance, PCT was not a significant predictor within 28 days of adverse medical outcomes in women with APN treated in the ED (21). Other studies have determined that both PCT and CRP are not useful in selecting the site for treating adult patients with APN (22). We thus investigated the predictability of PCT on septic shock, in the limited criterion of infected urolithiasis, as an attempt to minimize the effect of heterogeneous disease entities.

Second, we estimated the optimal cutoff value of serum PCT level to distinguish the patient at risk of septic shock from APN associated with urolithiasis, maximizing sensitivity without a substantial loss of specificity. The elevations of PCT are not as specific for infection as was once believed. For instance, mechanical trauma causes elevated PCT levels, the degree of which depends on the severity of the injury (23). Indeed, the PCT cut-off used to determine the risk of sepsis appears to be higher in critically ill patients admitted to intensive care units from the surgical service than in those admitted from the medical service (24). Therefore, one common cut-off for the diagnosis of septic shock is probably not feasible (6). Similar to our findings, expanding the clinical implication of PCT for specific condition, Nieuwkoop et al suggested different PCT cut-off level of 0.25μg/L as a robust surrogate marker for bacteremia in febrile UTI (14), just a half level different from our suggestion of 0.52μg/L as a marker for septic shock from urolithiasis.

Third, the peculiarity of this study is that we focused on the initial level of PCT. This study targeted patients presenting to an ED, so the analysis was based on the initial sampling of the ED presentation (within 1 hour after visit) rather than the peak levels or the changes of PCT after admission. Several studies that monitored PCT levels over time demonstrated that a policy looking for trends may be more predictive than the single initial level on admission (9, 10). In contrast, our data demonstrated that initial PCT levels at ED can provide additional aid to clinicians in their decision on the patient with infected urolithiasis. Given the increasing prevalence of sepsis associated urolithiasis, there is a clear need for a reliable diagnostic indicator that allows early discrimination between patients suffering from sepsis and those with septic shock. We believe that this approach using only the initial level of PCT is closer to the actual clinical setting, since in severely ill patients the presence of significant infection cannot always be specified. Similar to us, Hausfater et al. demonstrated the usefulness of PCT level in the ED to distinguish between mild-to-moderate sepsis and severe sepsis (25).

The strength of this study is based on the fact that the present study included consecutive unselected patients, and all available biochemical markers were measured at the same time by the same protocol. However, at the same time, we recognize the limitation of this study, besides of the small number of patients from a single institution. First, while the increasing body of literature supports the recently introduced notion that the course of PCT concentrations is a mirror of the systemic inflammatory response and is predictive for prognosis, PCT was not monitored along with the clinical course. Second, all data were collected prospectively, but the study design was not based on randomization of cases but on case-control series. Third, although our results may expand the role of PCT in controlling febrile UTIs, the cut-offs from these data cannot be adapted into other clinical situation. No single biomarker of sepsis or septic shock may be ideal, but the interest on the biomarkers as diagnostic and prognostic predictors in infectious disease is increasing, mainly because they reflect the host's response and predict disease progression (26). To expanding the role of PCT in various inflammatory situations, further studies with proper design are required.

## Conclusions

Our study demonstrated elevated initial PCT levels as an early independent predictor of progress into septic shock in patients with sepsis from APN associated with ureteral calculi. With a cut-off of 0.52ng/mL, reliable specificity and acceptable sensitivity can be obtained to distinguish the patient at high risk of septic shock. Based on these, we suggest PCT levels guide clinicians to identify the patient at risk of sepsis in the ED for infected ureteral calculi.

## References

[1] Curns AT, Holman RC, Sejvar JJ, Owings MF, Schonberger LB (2005). Infectious disease hospitalizations among older adults in the United States from 1990 through 2002. Arch Intern Med.

[2] Rautakorpi UM, Lumio J, Huovinen P, Klaukka T (1999). Indication-based use of antimicrobials in Finnish primary health care. Description of a method for data collection and results of its application. Scand J Prim Health Care.

[3] Hsu CY, Fang HC, Chou KJ, Chen CL, Lee PT, Chung HM (2006). The clinical impact of bacteremia in complicated acute pyelonephritis. Am J Med Sci.

[4] Faro S (1992). New considerations in treatment of urinary tract infections in adults. Urology.

[5] Foxman B, Klemstine KL, Brown PD (2003). Acute pyelonephritis in US hospitals in 1997: hospitalization and in-hospital mortality. Ann Epidemiol.

[6] Faix JD (2013). Biomarkers of sepsis. Crit Rev Clin Lab Sci.

[7] Meisner M, Tschaikowsky K, Palmaers T, Schmidt J (1999). Comparison of procalcitonin (PCT) and C-reactive protein (CRP) plasma concentrations at different SOFA scores during the course of sepsis and MODS. Crit Care.

[8] Matthaiou DK, Ntani G, Kontogiorgi M, Poulakou G, Armaganidis A, Dimopoulos G (2012). An ESICM systematic review and meta-analysis of procalcitonin-guided antibiotic therapy algorithms in adult critically ill patients. Intensive Care Med.

[9] Brunkhorst FM, Al-Nawas B, Krummenauer F, Forycki ZF, Shah PM (2002). Procalcitonin, C-reactive protein and APACHE II score for risk evaluation in patients with severe pneumonia. Clin Microbiol Infect.

[10] Bele N, Darmon M, Coquet I, Feugeas JP, Legriel S, Adaoui N (2011). Diagnostic accuracy of procalcitonin in critically ill immunocompromised patients. BMC Infect Dis.

[11] Eaton SH, Cashy J, Pearl JA, Stein DM, Perry K, Nadler RB (2013). Admission rates and costs associated with emergency presentation of urolithiasis: analysis of the Nationwide Emergency Department Sample 2006–2009. J Endourol.

[12] Bone RC, Balk RA, Cerra FB, Dellinger RP, Fein AM, Knaus WA (2009). Definitions for sepsis and organ failure and guidelines for the use of innovative therapies in sepsis. The ACCP/SCCM Consensus Conference Committee. American College of Chest Physicians/Society of Critical Care Medicine. 1992. Chest.

[13] Annane D, Bellissant E, Cavaillon JM (2005). Septic shock. Lancet.

[14] van Nieuwkoop C, Bonten TN, van't Wout JW, Kuijper EJ, Groeneveld GH, Becker MJ (2010). Procalcitonin reflects bacteremia and bacterial load in urosepsis syndrome: a prospective observational study. Crit Care.

[15] Trinchieri A (2008). Epidemiology of urolithiasis: an update. Clin Cases Miner Bone Metab.

[16] Sammon JD, Ghani KR, Karakiewicz PI, Bhojani N, Ravi P, Sun M (2013). Temporal trends, practice patterns, and treatment outcomes for infected upper urinary tract stones in the United States. Eur Urol.

[17] Bouadma L, Luyt CE, Tubach F, Cracco C, Alvarez A, Schwebel C (2010). Use of procalcitonin to reduce patients'exposure to antibiotics in intensive care units (PRORATA trial): a multicentre randomised controlled trial. Lancet.

[18] Ipek IO, Sezer RG, Senkal E, Bozaykut A (2012). Relationship between procalcitonin levels and presence of vesicoureteral reflux during first febrile urinary tract infection in children. Urology.

[19] Müller B, Becker KL, Schächinger H, Rickenbacher PR, Huber PR, Zimmerli W (2000). Calcitonin precursors are reliable markers of sepsis in a medical intensive care unit. Crit Care Med.

[20] Suprin E, Camus C, Gacouin A, Le Tulzo Y, Lavoue S, Feuillu A (2000). Procalcitonin: a valuable indicator of infection in a medical ICU?. Intensive Care Med.

[21] Lemiale V, Renaud B, Moutereau S, N'Gako A, Salloum M, Calmettes MJ (2007). A single procalcitonin level does not predict adverse outcomes of women with pyelonephritis. Eur Urol.

[22] Claessens YE, Schmidt J, Batard E, Grabar S, Jegou D, Hausfater P (2010). Can C-reactive protein, procalcitonin and mid-regional pro-atrial natriuretic peptide measurements guide choice of in-patient or out-patient care in acute pyelonephritis? Biomarkers In Sepsis (BIS) multicentre study. Clin Microbiol Infect.

[23] Wanner GA, Keel M, Steckholzer U, Beier W, Stocker R, Ertel W (2000). Relationship between procalcitonin plasma levels and severity of injury, sepsis, organ failure, and mortality in injured patients. Crit Care Med.

[24] Clec'h C, Fosse JP, Karoubi P, Vincent F, Chouahi I, Hamza L (2006). Differential diagnostic value of procalcitonin in surgical and medical patients with septic shock. Crit Care Med.

[25] Hausfater P, Garric S, Ayed SB, Rosenheim M, Bernard M, Riou B (2002). Usefulness of procalcitonin as a marker of systemic infection in emergency department patients: a prospective study. Clin Infect Dis.

[26] Drozdov D, Thomer A, Meili M, Schwarz S, Kouegbe RB, Regez K (2013). Procalcitonin, pyuria and proadrenomedullin in the management of urinary tract infections--'triple p in uti': study protocol for a randomized controlled trial. Trials.

